# Epigenetics of Inflammatory Bowel Diseases

**DOI:** 10.5152/tjg.2023.22515

**Published:** 2023-05-01

**Authors:** Murat Törüner, Nalan Gülşen Ünal

**Affiliations:** 1 Department of Gastroenterology, Ankara University Faculty of Medicine, Ankara, Turkey; 2 Department of Gastroenterology, Ege University Faculty of Medicine, İzmir, Turkey

**Keywords:** Inflammatory bowel diseases, ulcerative colitis, Crohn’s disease, epigenetics

## Abstract

Inflammatory bowel diseases are multifactorial, chronic, continuous, relapsing, and immune-mediated diseases of the gastrointestinal tract. It has been believed that mechanisms underlying inflammatory bowel diseases include genetic predisposition, environmental factors, and altered immune response to the gut microbiome. The epigenetic modulation takes place via chromatin modifications, including phosphorylation, acetylation, methylation, sumoylation, and ubiquitination. The methylation levels of colonic tissue were found well correlated to blood samples in inflammatory bowel diseases. Moreover, the methylation level of specific genes was different between Crohn’s disease and ulcerative colitis. It has been shown that the enzymes affecting histone modifications like histone deacetylases and histone acetyltransferases do not act solely on histones but also affect the acetylation of many proteins such as p53 and STAT3. It has been already shown that a nonselective histone deacetylase inhibitor, Vorinostat (SAHA), which is currently being used in several cancer treatments, showed anti-inflammatory activities in mouse models. Among epigenetic alterations, long non-coding RNAs and microRNAs play significant roles in T-cell maturation, differentiation, activation, and senility. The long non-coding RNA and microRNA expression profiles can perfectly separate inflammatory bowel disease patients from healthy controls and are remarked as biomarkers of inflammatory bowel diseases. Overall, many studies have shown that epigenetic inhibitors can target significant signal pathways in the pathogenesis of inflammatory bowel diseases, and the impact of epigenetic inhibitors is being studied in clinical trials. In conclusion, exploring more epigenetic pathways regarding inflammatory bowel disease pathogenesis will help us to discover therapeutic targets and new drugs and agents targeting miRNAs in inflammatory bowel diseases. In general, discovering epigenetic targets could improve the diagnosis and treatment of inflammatory bowel diseases.

## INTRODUCTION

Inflammatory bowel diseases (IBD), ulcerative colitis (UC), and Crohn’s disease (CD) are multifactorial, chronic, continuous, relapsing, and immune-mediated diseases of the gastrointestinal tract.^1^ Inflammatory bowel disease affects more than 1 million Americans, 2 million Europeans, and several hundreds of thousands of people from other parts of the world.^2-5^ Although it has been previously believed a disease in the western world, in recent years, incidence and prevalence of IBD have increased dramatically in developing countries and the eastern world including Turkey.^6,7^

It has been believed that mechanisms underlying IBD include genetic predisposition, environmental factors, and altered immune response to the gut microbiome.^8^ None of these underlying factors can explain the complex pathogenesis of IBD on their own. For example, in monozygotic twin studies, it has been identified that phenotypic concordance is only 50%-70% in CD and 10%-20% in UC.^9^ Only 2%-14% of the IBD patients have a family history of IBD.^10^ The first genetic locus on chromosome 16 has been described in 1996 as the first IBD-associated gene locus.^11^ This locus was characterized as the NOD-2 locus later on. Moreover, studies have shown that homozygosity of NOD2 is related to 20- to 40-fold increase in CD risk, whereas heterozygosity is only related to 2- to 4-fold increase in risk.^12,13^

To date, approximately 240 susceptibility loci for IBD have been discovered, which individually contribute only a small percentage of the expected heritability in IBD.^14^ There are several reports from European countries demonstrating that immigrants from lower-incidence countries such as India to higher-incidence countries such as the United Kingdom and Sweden are having a higher risk to have IBD than those from countries they migrated from.^15-17^ A study from Sweden showed that the increased risk of having IBD is more eminent in second-generation immigrants than in first-generation immigrants.^15^ Since genetic factors do not explain the whole pathogenesis underlying IBD and these epidemiological studies emphasize that environmental factors play some key roles as well as genetic factors in IBD pathogenesis, there should be a network and cross-talking between genetics and environmental factors.

Once it has been believed that the adaptive immune system has played the main role in IBD pathogenesis, recent studies showed that the innate immune system is playing an important role in inducing gut inflammation.

Several studies showed that altered host microbiota plays a role in IBD pathogenesis, and it is believed that the composition and diversity of the gut microbiota might be playing a crucial role in the development of IBD. Patients with IBD have dysbiosis in their luminal bacteria, which is mostly a reduction in the diversity of the microbial luminal community.^18-20^ It has been also shown that there is a greater fluctuation in gut microbiota compensation in IBD patients when compared to the normal population.^21^ The dysbiosis of gut microbiota is found to be greater in patients with CD than in patients with UC. Besides, data from twin studies showed that gut microbiota is different between healthy siblings and discordant twins.^22^ In IBD, studies showed that there is an alteration in gut flora.^23,24^ There are several reports demonstrating that such alterations might be correlated with the relapse of CD after ileal resection. Moreover, a cross-sectional study showed that *Faecalibacterium Prausnitzii* was reduced in patients with UC and their first-degree relatives as compared to normal controls.^25^ In the same study, the authors stated that lower *F.* *Prausnitzii* numbers are correlated with shorter remission periods and more frequent relapses/year. When patients are followed up, *F.* *Prausnitzii* numbers increased steadily to normal levels when patients were kept under remission and decreased in periods with relapse.

Like intestinal microbiota, environmental factors such as dietary habits and smoking play their role in IBD pathogenesis. Such environmental factors are found to be exerting their effect on intestinal microbiota resulting in a change in microbial composition^26^ and also they are shown to change the genome activity without changing DNA structure.^27^ Poor dietary habits can lead to the modulation of immune responses which might result in the alteration of inflammatory responses.^28^

In summary, IBD might be caused by interactions between the genome, alterations in the immune system, intestinal microbiota, and environmental factors such as diet and smoking. However, there are still question marks on which mechanisms play roles in such interactions. Possible answers to these can be explained by the field of epigenetics, which is currently an expanding and promising field of research area. This review insights into potential avenues and epigenetic modifications in IBD.

## Description of Epigenetic Alteration

The gap between the environmental factors underlying autoimmune disorders and genetic susceptibility was filled by epigenetic mechanisms. Epigenetics is the study of mitotically heritable changes in the genome function without a change in nucleotide sequence. The main epigenetic mechanisms are DNA methylation, histone acetylation, RNA interference, and the positioning of nucleosomes. These epigenetic mechanisms play an important role in the interaction between genome and the environment. They are potentially heritable and reversible through cell division.^29^ Through these epigenetic processes, cells might be differentiated into different cell types and new phenotypes can be transferred to daughter cells.^30^

Disorders in epigenetic mechanisms (DNA methylation, histone acetylation, and non-coding RNA expression) can be linked to autoimmune disorders. DNA methylation is established and maintained by DNA methyltransferase (DNMT) enzyme family including DNMT 1, DNMT 3a, DNMT 3b, and DNMT 3L while DNA demethylation can be established through passive demethylation by inhibition on methylation or active demethylation by enzymes called cytosine deaminases. DNA methylation occurs commonly on the 5ʹ position on pyrimidine cytosines, which is catalyzed by DNMTs. DNA methyltransferasess are both responsible for maintaining DNA methylation (DNMT1) and de novo methylation (DNMT 3a/b).^31^ Chromatin is the combination of both DNA and proteins, especially histones, which organize DNA into structural units, called a nucleosome. Chromatin state can be modified by DNA methylation as well as post-transcriptional modifications of histones, which are the second major epigenetic mechanism.

Histones can be modified through acetylation, methylation, phosphorylation, or ubiquitination, which occur commonly in histone tail regions. Histone post-transcriptional modifications are usually kept in balance with histone methyltransferases and histone demethylases, histone acetyltransferases (HATs), and histone deacetylases (HDACs). Histone acetylation by HAT usually results in promoting a more open chromatin structure. Generally, hyperacetylation is characteristic of active genes. In contrast, histone deacetylation catalyzed by HDAC promotes a well tightly wrapped nucleosome structure and represses gene expression, finally resulting in gene silencing.^32^

microRNAs (miRNAs) are single-stranded non-coding RNAs and usually 22 nucleotides in length. Since their first description in the 1990s, over 1500 miRNAs have been described. Through binding on functional RNAs, miRNAs can directly inhibit mRNA translation or induce mRNA degradation.^33^ It has been shown that each miRNA can show complementarity with more than one mRNA, whereas each mRNA can be regulated by more than one miRNA.^34,35^ Altered miRNA expressions were found to be many neoplastic diseases and non-neoplastic diseases.

Nowadays, it is becoming clear that there is crosstalk between different epigenetic mechanisms as well as between epigenetic mechanisms and environmental factors. There are several studies which showed that DNA methylation can affect histone acetylation or the opposite.^36,37^ It has also been shown that a maternal methyl donor supplementation diet can result in DNA hypermethylation and induce colitis in offspring murine.^38^

## Inflammatory Bowel Diseases and Epigenetic Alteration

In recent years, in the field of autoimmune diseases, a lot of progress has been achieved in detailed epigenetic mechanisms to fill the gap between environmental factors and genetic background. Disorders in epigenetic processes such as DNA methylation and histone modifications have been identified in autoimmune diseases’ pathogenesis. For example in rheumatoid arthritis, DNA hypomethylation has been shown in peripheral blood mononuclear cells,^38^ whereas DNA hypermethylation has been shown in synovial fibroblasts.^39^ Concerning tissue samples, it was shown that all hypermethylated genes exhibit the same methylation pattern. Furthermore, methylation levels of colonic tissue were found well correlated to blood samples in IBD patients. Moreover, the methylation level of specific genes was different between CD and UC. Although CXCL14, CXCL5, GATA3, IL17C, and IL4R were hypermethylated in UC compared to CD, IL13 was hypermethylated in CD compared to UC. Therefore, methylation levels may be used as a non-invasive marker to diagnose and differentiate CD from UC in the future.^40^

Through epigenetic mechanisms, environmental factors like nutrition and smoking have shown that they can exert their effects on the pathogenesis of autoimmune diseases such as IBD, diabetes mellitus, and rheumatoid arthritis. The expression levels of the significant gene pathways for IBD may be modulated by dietary intervention. Macro- and micronutrients (folate, vitamin B_12_, vitamin D, selenium, and zinc), polyphenols, and phytochemicals may affect DNA methylation, HDAC inhibition or activation, and the gut microbiota, thereby epigenetic silencing of genes. Vitamin D receptor (VDR) gene polymorphisms are increasingly recognized as important in IBD pathogenesis.^41^

A recent study demonstrated that ring finger protein 20/ring finger protein 40 (RNF20/RNF40) was epigenetic modifier responsible for histone protein 2B (H2B) monoubiquitination. Loss of H2B monoubiquitination promotes intestinal inflammation via decreased VDR activity in CD patients and mouse models. Nevertheless, all these findings supporting an important role of vitamin D and VDR polymorphisms in IBD, environmental, or epigenetic modifiers are remained to be elucidated.^42^

Smoking is a well-known factor that causes widespread epigenetic changes linked with an increased risk of smoking-related diseases and elevated mortality. Smoking induces intestinal inflammation by changing the intestinal epithelium, immune system, microbiota functions, and interactions.^43^ A recent study pointed out that miR-124 mediated the epigenetic effect of nicotine in IBD by shifting the Th1/Th2 balance toward Th1. They demonstrated that overexpressed miR-124 protected against mouse dextran sodium sulfate (DSS)-induced colitis with a Th1 polarization in peripheral blood lymphocytes and colon tissues, as well as in human peripheral blood lymphocytes.^44^

As mentioned above, IBD is a disease group with an unknown exact etiology and the pathogenesis of IBD is believed to involve genetic background, environmental factors, and alterations in the host immune system. Thus, epigenetic mechanisms underlying IBD might help us to understand the exact mechanism of the disease and to fill the gap between other underlying mechanisms. Previous studies showed that there was an increased risk of developing colorectal cancer in both UC and Crohn’s colitis compared with the general population.^45^ Therefore, initial epigenetic studies investigated the relationship between long-term IBD and colon cancer. The main epigenetic mechanism underlying colitis-associated colorectal cancer was reported as DNA hypermethylation.^46^ DNA methylation in colonic epithelial cells normally occurs in aging; however, due to inflammation and high cell turnover, increased DNA methylation is observed in IBD as well.^47^ It has also been shown that increased DNA methylation is observed more in dysplastic colon tissues when compared with ones with no dysplasia.^47^ Also, it has been found that 4 out of 15 loci (CDH1, GDNF, HPP1, and MYOD1), which are associated with cancer development, are highly methylated in colonic tissue samples from active UC patients when compared to the samples from quiescent UC patients.^48^ Hypermethylation of CDH1 (that encodes cell adhesion molecule, E-cadherin, which is found to be associated with IBD-associated cancer) promoter region was shown in dysplastic tissue samples when compared with non-dysplastic tissue samples from UC patients.^49^

Following these first epigenetic studies regarding IBD and colorectal cancer, the first epigenome-wide association studies were established in 2012. These studies evaluated peripheral blood from children and women with IBD for the methylation profile and obtained 50 genes including MAPK, RPIK3, and IL21R, which are involved in immune system activation show significantly different methylation levels when compared to normal controls.^50^

## The Epigenetic Mechanism of T Cells and B Cells in Inflammatory Bowel Disease

Cancer development-related signaling pathways such as PI3K-AKT, Ras, Wnt, and TGF-beta were found enriched in the colitis tissue.^46^ In a recent study, it was shown that Wnt-β-catenin activation alters the Treg cell population in IBD and dysplastic progression via epigenetic and transcriptional changes of Foxp3-TCF-1 co-regulated genes. That is, this mentioned study has verified progressive and systemic expansion of RORγt^+^ Treg immune cells during the IBD course and progression to dysplasia with epigenetic modifications.^51^

Another study analyzed DNA methylation in Epstein-Barr Virus (EBV) transformed B cells from IBD patients and found 49 differentially methylated CpG sites. More than half of these sites are related to the regulation of the immune system and IL-12/IL23 pathways such as BCL3, STAT3, STAT 5, and OSM.^51^ Furthermore, another methylation-wide study using isolated rectal epithelial cells from active/quiescent UC and CD patients identified differentially methylated genes including DOK2, Tap1, and TNFSF4/12. In the same study, ULK1 which has a role in autophagy was found to be methylated only in CD.^52^

**Figure 1.**
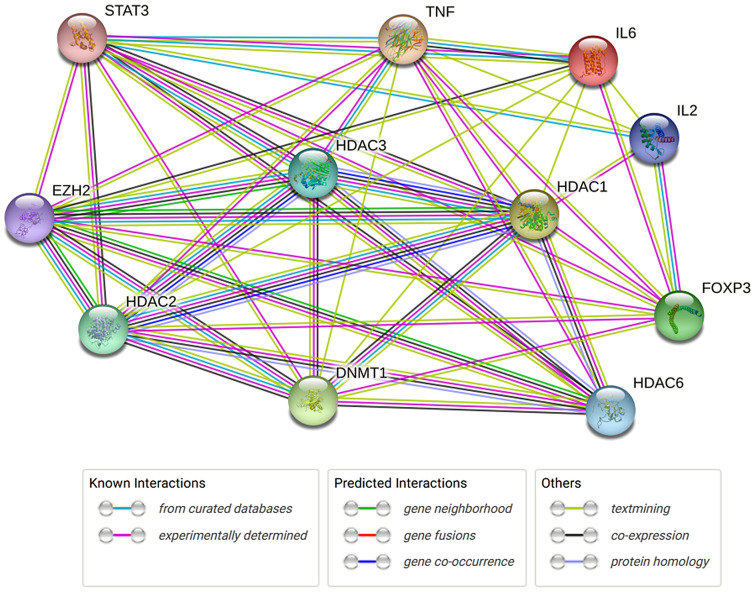
The String network analysis epigenetic related genes and inflammatory associated genes.

A very recent study evaluated the methylation patterns in intestinal epithelial cells as well as gut microbiota patterns in pediatric patients with IBD.^53^ The authors studied differentially methylated positions, differentially expressed genes (DEGs), and regulatory differentially methylated regions (rDMRs) in colonic epithelial cells derived from different parts of the intestine from IBD (UC and CD) patients and control subjects. When comparing identified DMRs, DEGs, and others, there were significant changes between IBD and the control group. Also, DNA methylation profiles from the same patient at 2 different time points are evaluated and it is found that disease-associated methylation signatures did not change by medication changes or inflammation status. When investigators clustered the data from DNA methylation and gene expression, they demonstrated a significant difference in outcomes of the patients depending on DNA methylation and gene expression.^53^ Here, we analyzed the protein–protein interactions (epigenetic-related genes and inflammatory-associated genes) utilizing the String database (https:// string-db.org/) (Table 1 and Figure 1).

Histone modifications have been shown to regulate genes that affect chronic inflammation.^54^ Since CD4^+^ T cells are playing a crucial role in the pathogenesis of autoimmune disorders like multiple sclerosis and CD^55^ and are believed to be one of the key regulators of intestinal inflammation,^56^ there have been several publications that investigated the epigenetic alterations in CD4^+^ T cells. When induced with antigens and cytokine networks, naïve CD4+ T cells are converted into different phenotypes such as Th1, Th2, Th17, and Treg.^57^ Treg cells are defined by the expression of a specific transcription factor called FOXP3, which is playing a unique role in immune system regulations.^58^ Moreover, humans with FOXP3 mutations develop autoimmune disorders like polyendocrinopathy and enteropathies.^59,60^ EZH2 is a histone methyl transferase that interacts with FOXP3 target genes in immune regulatory pathways. Investigators from Mayo Clinic have shown that EZH2 is recruited to the silenced FOXP3 promoter through a polycomb response element.^61^ The same group of investigators from Mayo Clinic have also shown that EZH2 deficiency in FOXP3^+^ cells in mice resulted in multiorgan immunity and decreased survival.^62^ These mice developed spontaneous IBD. As gene expression networks of CD4+ T cells extracted from intestines of IBD patients indicated disrupted EZH2 networks and differential expression of proinflammatory genes typical of Th1/Th17 effector T cells, one can claim that deregulation of EZH2-enforced T-cell gene networks leads and maintains intestinal inflammation in both murine models and human IBD.

Much of the knowledge about the role of histone modifications on inflammation comes from the experimental trial of histone deacetylase inhibitors (HDACi), which are being investigated for cancer treatments. Histone deacetylase inhibitors are classified according to their structural class including carboxylates, hydroxamic acids, and cyclic peptides.^63^ It has been shown that the enzymes affecting histone modifications like HDACs and HATs do not act solely on histones but also affect the acetylation of many proteins such as p53 and STAT3.^64^ Therefore, they can act through the epigenetic mechanisms and other non-epigenetic mechanisms as well. Histone deacetylases contain 18 isoforms that are divided into 4 groups, class I, class II, class III, and class VI having different functions.^65^ It has been already shown that a nonselective HDAC inhibitor, Vorinostat (SAHA), which is currently being used in several cancer treatments showed anti-inflammatory activities in the mouse models.^66^

Several other studies supported the idea of using HDACi as a possible treatment option for IBD. It has been shown that the administration of HDACi to mice leads to a decrease in intestinal inflammation as a result of acetylation of FOXP3.^67^ In 2011, De Zoeten et al^68^ showed that Tubacin, which is a selective HDAC6 inhibitor, inhibited intestinal inflammation in a DSS-induced colitis model through its effect on FOXP3^+^ Treg cells. In addition, selective HDAC9 inhibition increases the function of FOXP3 Tregs and ameliorated mice colitis.^69^

Following these studies, recent 2 studies have demonstrated that HDACi decreased the levels of inflammation in a mouse IBD model, dextran sulfate sodium-induced colitis. One of them showed that a potent HDACi, BML-281, suppressed the infiltration of CD19^+^ B cells into the colonic epithelial cells, and this suppression led to decreased inflammation in the colonic mucosa.^70^ In the second study, the authors used LTB2 which is an HDAC-6 inhibitor (class II HDAC) as a treatment model in the DSS-induced colitis model in mice. In the LTB2-treated group, the authors showed a decreased inflammation histologically and decreased clinical aspects such as rectal bleeding and diarrhea.^71^

## Studies of Epigenetic Changes as Biomarkers in Inflammatory Bowel Disease

### Long Non-coding RNAs and Inflammatory Bowel Disease

**Figure 2.**
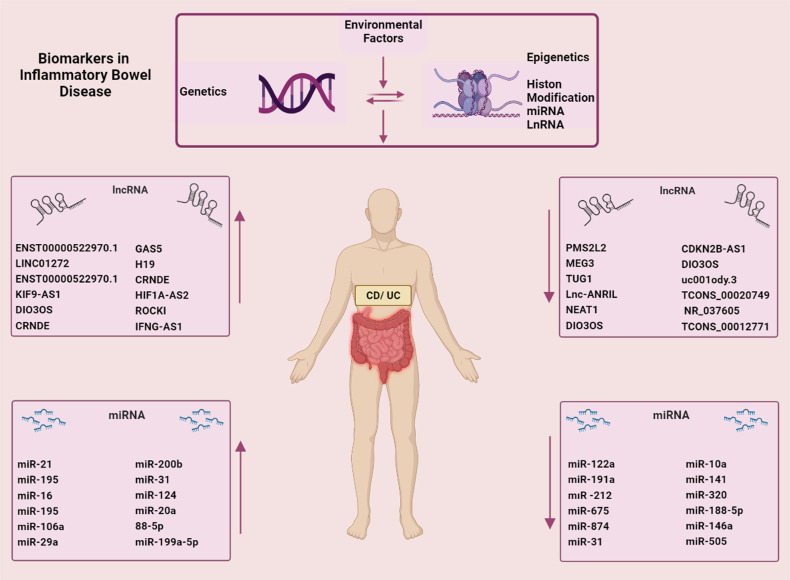
The different factors and biomarkers of epigenetics in IBD.

Long non-coding RNAs with >200 nucleotides length are a new group of non-coding RNAs that are related to inflammation and can modulate gene expression via post-transcriptional regulation, transcriptional regulation, and chromatin modifications. Dysfunction or dysregulated expression of numerous lncRNAs has been noticed to initiate and promote inflammatory diseases. The studies indicated that lncRNAs such as KIF9-AS1, LINC01272, and DIO3OS as new biomarkers had roles in diagnosis for the detection of IBD^72-74^ (Figure 2).

The overexpression of lncRNA MEG3 reduces ulceration through upregulating IL-10 by sponging miR-98-5p and is a therapeutic strategy for UC treatment.^75^ The animal studies demonstrated that overexpression of TUG1 decreased UC progression. Also, overexpression of TUG1 promoted cell proliferation and attenuated cell apoptosis in the TNFα-triggered cells.^76^

Long non-coding RNA antisense non-coding RNA in the INK4 locus (lnc-ANRIL) has been described to be related to inflammation and immunity. However, few studies have disclosed the relationship between lnc-ANRIL and proinflammatory cytokines in severe or moderate IBD. The studies have referred to its clinical use in pediatric IBD diagnostics and its application as a major marker for appraising disease risk in pediatric IBD patients.^77^ The lncRNA expression profile can perfectly separate IBD patients from healthy control individuals. The clinically relevant parameters and transcription characteristics of lncRNA remark that lncRNAs are biomarkers of IBD.

### microRNAs and Inflammatory Bowel Disease

**Figure 3.**
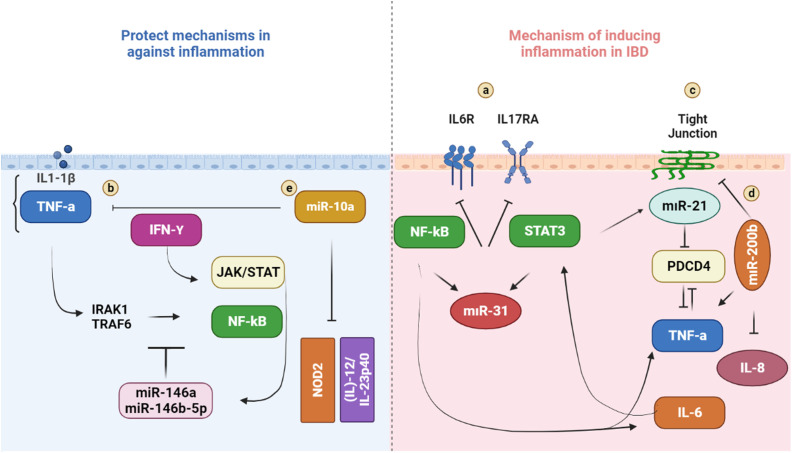
miRNAs-associated with several pathophysiological factors of IBD.^88-92^

microRNAs are endogenous, single-stranded, non-coding RNA and have roles in the post-transcriptional regulation of gene expression.^78^ The expression of miRNAs that appears importantly dysregulated would cause inhibition or activation of signaling pathways and lead to types of diseases such as cancer and inflammatory disease^79^ (Figures 2 and 3).

As mentioned above, RNA interference and miRNAs are the third major mechanism in epigenetics. Studies in animals demonstrated that miRNAs regulate gut hemostasis, and in recent years, there are several papers published regarding tissue and peripheral blood miRNA expression profiles and their functional importance in IBD pathogenesis. Several studies identified specific miRNAs regulating several IBD-associated genes such as NOD2, IL23, and ATG16L1.^80,81^ Distinct miRNA signatures have been identified in peripheral blood samples from CD and UC patients when compared to healthy subjects and quiescent IBD patients.^82-84^ In addition, 11 miRNAs were also found to be differently expressed between pediatric IBD patients and healthy children.^85^ miRNA-106a and -106b are members of the miRNA-17 family, which are one of the first reported miRNAs to be differentially expressed in IBD patients. The miRNA-17 family is now known to target multiple autophagy genes that are associated with IBD, including ATG16L1. It has been demonstrated that miRNA-106b binds ATG16L1 resulting in decreased autophagy.^86^ Furthermore, another group of investigators showed that increased expression of miRNA-106b is correlated with downregulated ATG16L1 in patients with active CD.^80^ Recently, it has been demonstrated that multiple miRNAs are involved in the regulation of NOD2 gene expression and NOD2 is not only regulated by miRNAs but also exerts its downstream effects via miRNAs. Brain et al showed that miRNA-29 downregulates IL-23 production by targeting its subunit p40.^81^ It is also known that dendritic cells from NOD2 homogeneous or heterogeneous CD patients are associated with failure to induce miRNA-29 resulting in increased production of IL23, which is currently a clinical therapeutic target in CD therapy. Following the discovery of miRNA dysregulation in IBD and in the light of preliminary studies, further studies have been developed to investigate potential miRNA-based therapies. In recent years, there is an increasing number of studies coming out in the field of possible clinical applications of miRNA-targeted therapies in IBD. For instance, miRNA-301a levels were elevated in TNBS-induced colitis; however, if miRNA-301a inhibitors are applied as an enema to mice with colitis, there is a decrease in proinflammatory cytokines that reflects decreased inflammation.^87^ In another example, investigators showed that by inhibiting miRNA210, which is also found to be highly expressed in the colonic mucosa of UC patients, they have been able to decrease the levels of inflammation in TNBS-induced colitis in mice.^88^

### Epigenetic Inhibitors

IL-12p35 and EBV-induced gene 3 (EBI3) are members of the IL-35 family, and they have an anti-inflammatory role in UC. EBI3 was recently demonstrated to be a target for nuclear factor kappa-B (NF-κB) or nuclear factor kappa-light-chain-enhancer of activated B cells in human colon epithelial cells. Also, it has been shown that EBI3 expression is increased by epigenetic histone acetylation in human colon epithelial cells. The treatment with either the HDACi or the proinflammatory TNFα upregulates EBI3 expression in the colon epithelium.^89^

**Figure 4.**
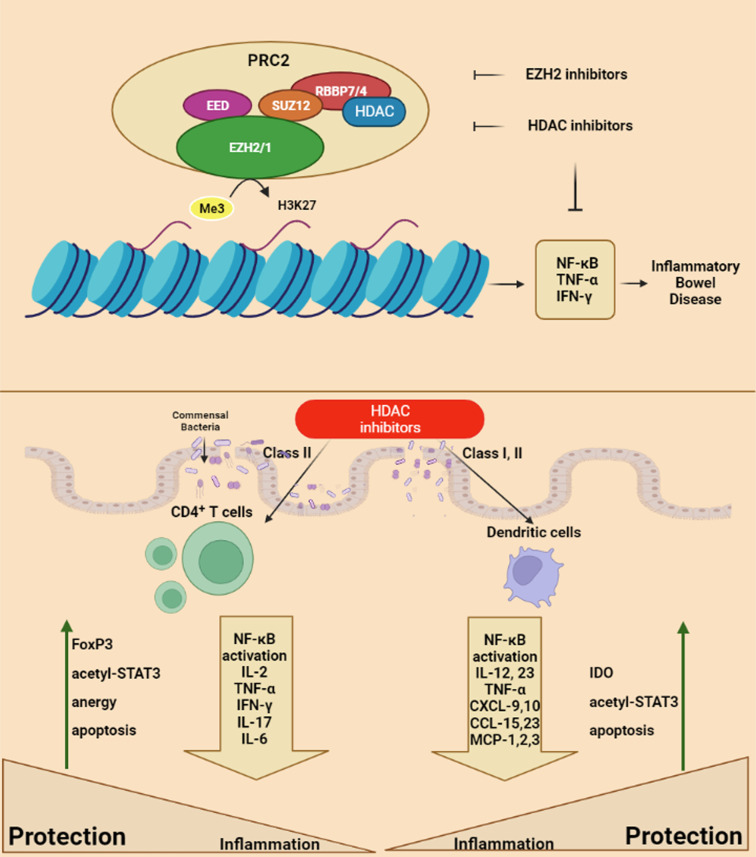
HDAC and EZH2 inhibitors regulate inflammatory bowel diseases.

Zeste homolog 2 (EZH2) is an element of polycomb-repressive complex 2 (PRC2). EZH2 initiates the histone H3 methylation at lysine 27 and has roles in various epigenetic modifications, proliferation, adhesion, cell differentiation, and survival. The role of EZH2 is evident in regulating primary immune cells such as T cells and B cells. EZH2 has a pathogenic role in several particular autoimmune diseases including rheumatoid arthritis, type I diabetes, systemic lupus erythematosus, multiple sclerosis, and IBD. EZH2 has a role in UC development. But, the mechanism of EZH2 in UC progression is unknown. Regarding the TNFα-mediated NF-κB pathway, the reduction of EZH2 hypothesized the participated effects of EZH2 on NF-κB signaling in enhancing inflammation in IBD patients^90-92^ (Figure 4).

Dysfunction of forkhead box protein 3 (FOXP3)^+^ regulatory T cell is related to IBD. The functional and physical interaction between (FOXP3)^+^ and EZH2 is essential for Treg cell function. The mutation in FOXP3 cysteine 232 (C232) has demonstrated disrupted suppression of IL-2 and reduced EZH2-mediated H3K27me3 on IFN-γ, indicative of compromised physiologic functions of Treg cells. In human Treg cells, IL-6 abolishes FOXP3-EZH2 interaction, via an increase in post-translational modification of FOXP3. Also, this recent study has shown that the FOXP3-EZH2 protein complex suppresses immunity, and is referred to the therapeutic implication of stabilizing FOXP3-EZH2 interaction to recovering Treg function in IBD^93^ (Figure 4).

EZH2 inhibition could repress sepsis by reducing the inflammatory response and pulmonary cell apoptosis via repression of the signal transduction pathway and STAT3 signaling and stimulation of peroxisome proliferator-activated receptor-γ (PPARγ).

Angiopoietin-like 4 (ANGPTL4) plays an anti-inflammatory role in UC. ANGPTL4 decreases the inflammatory response by regulating the NF-κB pathway in the DSS-induced colitis model. Additionally, ANGPTL4 could be regulated by EZH2 to accompany various cell signal pathways. The results of a study have demonstrated that the inhibition of EZH2 by GSK 343 reduced LPS-induced inflammatory response and cell apoptosis in the DSS-induced UC model, possibly through promoting ANGPTL4/CREB1.^94^

Also, the PRC complexes play a role in the regulation of the FOXP3 target gene. A study has demonstrated that BMI1, a PRC1 family member, has a role in the modulation of a proinflammatory enhancer network in CD. Further, the knock-out of BMI1 or using BMI1 inhibitor (PTC209) in murine FOXP3+ cells results in systemic inflammation. BMI1-missing Tregs carried a TH1/TH17-like phenotype as determined with proteomic techniques.^95^

Dendritic cells (DCs) play a fundamental role in promoting T-cell-mediated forbearance to self-antigens and trigger inflammation to safe antigens, and deletion of DCs triggers the development of spontaneous fatal autoimmune events. Oppositely, DCs could be critical for the stimulation of inflammatory phenotypes on T cells reactive to safe antigens in the initiating of inflammatory and autoimmune diseases such as IBD.^96,97^

GSK-J4 is an inhibitor of the histone demethylase JMJD3/UTX, which decreased DSS colitis. The mechanism of GSK-J4 is inhibition of H3K27me3 demethylase, and this effect is regulated by modifies on histone post-translational modifications at the RALDH1 and RALDH1 (retinaldehyde dehydrogenase) promoters in dendritic cells, which enhances RALDH activity in DCs, thus favoring the de novo retinoic acid (RA) synthesis. RA-mediated mechanisms can act in the generation of peripheral Treg-like CD4^+^ T cells. This triggers Treg with higher repressive function and stability and also enhanced gut-tropism, consecutively. Therefore, GSK-J4 (a KDM6A/6B inhibitor) has been suggested as a promising therapeutic strategy for IBD.^98,99^

The SWI/SNF complex modifies the chromatin structure of the regulatory sequences of inflammatory-related genes and makes induction of inflammation genes during the inflammation process such as the NF-κВ signaling pathway. The role of SWI/SNF in the initiation of cancer is well figured out and mutations in genes encoding subunits of SWI/SNF happen in 20%-25% of cancers. The SWI/SNF complex has roles in the modulation of acute inflammation, which can transform chronic inflammation, and appropriately result in onco-transformation of cells. The SWI/SNF roles in the control of the expression of inflammatory genes are still unknown. Therefore, studying the role of the SWI/SNF complex in the modulation of inflammation genes is significant for practical medicine.^100^

Bromodomain and extra-terminal motif (BET) proteins including BRD2, 3, 4, and BRDT are epigenetic “reader” proteins that bind to acetylated lysine residues on histone proteins. The inhibition of the BRD4 to acetylated histones has been demonstrated to repress the MYC oncogene expression and the proinflammatory cytokines, such as IL-6, IL-1β, IL-12α, and INF-β in murine models of IBD. BET inhibitor (CN210) inhibits the expression of inflammatory cytokines via the suppression of MAPK and NF-κB pathways. Therefore, BET inhibitors as epigenetic inhibitors represent a new treatment for IBD.^101^

Recently, many studies have shown that epigenetic inhibitors can target significant signal pathways in the pathogenesis of IBD, and the impact of epigenetic inhibitors is being studied in clinical trials. Molecular targeting of epigenetic changes and utilizing particular epigenetic modifications of IBD for diagnosis and treatment will be advantageous to the diagnosis, prevention, and treatment of IBD.

## CONCLUSION

Until now, the complete pathogenesis underlying IBD is not clear. Although genetics, environmental factors, intestinal microbiota, and immune system alteration are the ones blamed as the underlying factors, there is still an unfilled gap between those factors to establish a fully comprehendible pathogenesis model. To our understanding, the missing linkage between these possible underlying mechanisms might be explained by epigenetic mechanisms to some extent. Currently, studies have remarked that epigenetic factors, such as miRNA dysregulation, lncRNA dysregulation, and histone modifications, play crucial roles in the pathogenesis of IBD.

Epigenetic studies will further provide biomarkers to be used in the diagnosis and differential diagnosis of IBD. Moreover, exploring more epigenetic pathways regarding IBD pathogenesis will help us to discover therapeutic targets and new drugs such as HDACi and agents targeting miRNAs in IBD. In general, discovering epigenetic targets could improve the diagnosis and treatment of IBD.

## Figures and Tables

**Table 1.** Protein–Protein Interaction (PPI) Network Stats

Number of proteins	11
Number of edges	46
Average node degree	8.36
Avg. local clustering coefficient	0.896
Expected number of edges	16
PPI enrichment *P*-value	**.0000000003**
